# Antibiotic treatment following a dog bite in an immunocompromized patient in order to prevent *Capnocytophaga canimorsus* infection: a case report

**DOI:** 10.1186/1756-0500-7-432

**Published:** 2014-07-05

**Authors:** Ondrej Hloch, Dana Mokra, Jan Masopust, Jan Hasa, Jiri Charvat

**Affiliations:** 1 Metabolic Intensive Care Unit, Medical Department, 2nd Faculty of Medicine of Charles University and Faculty Hospital, Prague Motol, V Úvalu 84, 150 06 Prague 5, Czech Republic

**Keywords:** *Capnocytophaga canimorsus*, Sepsis, Multiorgan failure, Nosocomial infection

## Abstract

**Background:**

*Capnocytophaga canimorsus* is a commensal bacterium found in the saliva of dogs and cats. Clinically significant infections in humans after a bite are often associated with the presence of immune deficiency. Early recognition and appropriate treatment are crucial for patient survival. In addition, patients with immune deficiency are susceptible to serious life-threatening nosocomial infections, which may also influence the prognosis of patients with *Capnocytophaga canimorsus* infection.

**Case presentation:**

A 62-year-old Caucasian female was admitted with septic shock, acute respiratory distress syndrome, acute renal failure, metabolic acidosis and disseminated intravascular coagulation after suffering two small bites from her dog. She had received a splenectomy during childhood. The patient survived after early empiric treatment with antibiotics and intensive supportive care, including ventilation support, a high dose of noradrenalin, and continuous venovenous hemodialysis applied prior to the definitive diagnosis of *Capnocytophaga canimorsus* sepsis. She improved within 2 weeks but, despite all efforts to prevent nosocomial infection, her hospital course was complicated by *Enterococcus* species and *Candida albicans* pleuropneumonia that prolonged her stay in the intensive care unit, and necessitated ventilation support for 2 months.

**Conclusion:**

Severe *Capnocytophaga canimorsus* sepsis may be complicated by life-threatening nosocomial infection in immunocompromized patients. The prophylactic application of antibiotics after a dog bite should be considered in high-risk individuals with immune deficiency in order to prevent both *Capnocytophyga canimorsus* sepsis and serious nosocomial complications.

## Background

*Capnocytophaga canimorsus* is a commensal bacterium found in the saliva of 16–24% of dogs and 17% of cats [1,2]. It is a slowly growing Gram-negative bacillus that has been implicated in a variety of disorders including sepsis, meningitis, thrombotic thrombocytopenic purpura, and endocarditis [3-8]. The first case of the infection in humans was reported by Bobo and Newton in 1976 [9]. The source of infection is usually a dog (91%), though infection from cats has been reported in 9% [4,10,11]. *Capnocytophaga canimorsus* is known to have relatively low virulence, and clinically significant infections are associated with the presence of immune deficiency. The majority of cases have been described in splenectomy patients or in chronic alcoholics [12-14]. The mortality of patients suffering from *Capnocytophaga canimorsus* is very high in those suffering septic shock and multiorgan failure [10].

Patients with immune deficiency may develop nosocomial infections that further compromise their condition and may also be life-threatening. Thus far, we have not found in the literature any report of *Capnocytopaga canimorsus* sepsis complicated by *Enterococcus* species and *Candida albicans* nosocomial infections. We present such a case of a woman who had a splenectomy in childhood and was bitten by her dog. Our report is a contribution to the discussion of the prophylactic application of antibiotics in high-risk individuals with immune deficiency in order to prevent both *Capnocytophyga canimorus* sepsis and serious nosocomial complications.

## Case presentation

A 62-year-old Caucasian woman presented to the traumatology department with two small superficial wounds 2 × 2 mm after a bite from her dog, and without any signs of local infection (Additional file 1). The wounds were disinfected and covered with a sterile dressing, and she was sent home.

The patient had undergone a splenectomy when she was 10 years old, but she had no knowledge of the indication for this surgical procedure. Since then, she had been healthy and had never been admitted to hospital. Thirty six hours after the dog bite, she became febrile, had muscle discomfort, and had general weakness. She presented to the emergency department where her blood pressure was 100/45 mmHg and, apart from C-reactive protein (CRP) 9 mg/L, all biochemical and hematological parameters were within normal limits. After application of crystalloid infusion, her condition improved and she was discharged home. Her symptoms were attributed to a viral infection.

However, after another 2 days she was admitted to the metabolic intensive care unit with a clinical picture of septic shock. She was tachypneic, febrile, and had acrocyanosis and decreased capillary return. Her blood pressure was 70/57 mmHg, and an electrocardiogram revealed sinus tachycardia of 105–115 beats/minute. Her CRP level was 237 mg/L, procalcitonin was 3.18 μg/L, white blood cells were 30.9 × 10^9^/L, APACHE (Acute Physiology and Chronic Health Evaluation) score was 27, and SOFA (Sequential Organ Failure Assessment) score was 16 (Table 1).

**Table 1:** Clinical characteristics

	**0 h**	**24 h**
**Arterial pressure** [mmHg]	70/47	117/75
**Heart rate** [minˉ^1^]	114	72
**Temperature** [grade Celsius]	36,2	37
**Diuresis** [mL/h]	0	0
**Serum urea** [mmol/l]	8,9	16,8
**Serum creatinine** [μmol/l]	174	253
**pH**	7,23	7,25
**pCO2** [kPa]	6,66	5,42
**pO2** [kPa]	6,29	10,5
**HCO3ˉ** [mmol/l]	21,4	17,9
**INR**	1,65	1,8
**APTT** [s]	34,2	42,4
**C-reactive protein** [mg/L]	237	315
**Procalcitonin** [μg/l]	53,01	75,68
**White blood cells** [×10^9^/L]	2,5	18,9
**Hemoglobin** [g/L]	10,9	12,5
**Hematocrit**	0,317	0,373
**Platelet count** [×10^9^/L]	72	25
**APACHE II**	27	29
**SOFA**	16	21
**NT-proBNP** [ng/L]	20 506	

INR……………..international normalized ratio.

APTT……………activated partial tromboplastin time.

APACHE……….Acute Physiology and Chronic Health Evaluation.

SOFA……………Sequential Organ Failure Assesment.

NT-proBNP……..N-terminal pro-brain natriuretic peptide.

A peripheral three-ways central venous catheter (PICC) was introduced via the right brachial vein, and an arterial cannula placed in the right radial artery. PICC was selected because of abnormal coagulation. The hemodynamic response to volume expansion was insufficient, and the application of catecholamine support was necessary. Noradrenalin was given at a dose of 50 μg/min and gradually increased up to 66 μg/min in order to maintain the mean arterial blood pressure above 65 mmHg. The patient also presented with acute respiratory failure with arterial desaturation, carbon dioxide retention, and respiratory acidosis. She was sedated, intubated and put on artificial respiratory support. A high fraction of oxygen (80%) and positive expiratory pressure were necessary for efficient ventilation. Comparison of chest X-rays in the emergency room and another after admission demonstrated the speed of evolution of acute respiratory distress syndrome (ARDS) (Additional file 2). One week later a tracheostomy was carried out. Routine practice was implemented to prevent aspiration (30–45 degree head of bed elevation, regular subglottic suctioning, comprehensive oral care with antiseptic solution, etc.).

An empiric combination of broad-spectrum antibiotics (piperacilinum tazobactam + ciprofloxacin) was given as soon as blood samples were drawn for microbiological examination. Many units of fresh frozen plasma were given because the patient presented with disseminated intravascular coagulopathy and severe thrombocytopenia (24 × 10^9^/L) accompanied by petechiae (Additional file 3).

The patient developed acute kidney failure grade F according to the RIFLE (Risk, Injury, Failure, Loss of kidney function, and End-stage kidney disease) classification. As the hemodynamic situation of the patient was compromised, continuous venovenous hemodialysis (CVVHD) was selected for renal replacement therapy, and applied on the second day of admission in the intensive metabolic care unit. A hemodialysis catheter was inserted into the right femoral vein. Nutrition was provided by early enteral nutrition through a nasojejunal tube.

On the 5th day of admission, blood culture revealed massive infection with Gram-negative bacteria identified as *Capnocytophaga canimorsus*, which was resistant only to cotrimoxazol. According to the minimum inhibitory concentration, clindamycin 600 mg every 6 hours was added to the antibiotic regimen.

The clinical condition of the patient gradually stabilized. Noradrenaline application was stopped on the 9th day of admission and CVVHD was changed to intermittent hemodialysis on the 13th day of admission. Renal function steadily improved. Her fever resolved, and her vital functions improved, allowing her to be weaned from ventilation support. She was able to breathe spontaneously with peak end expiratory pressure 5 mmHg. Sedation was reduced and she communicated with nursing staff. Her CRP decreased to 30 mg/L, procalcitonin to 0.64 μg/L, white blood cells to 11.1 × 10^9^/L, platelet count normalized to 200 × 10^9^/L, and her chest X-ray was normal.

**Figure 1:**
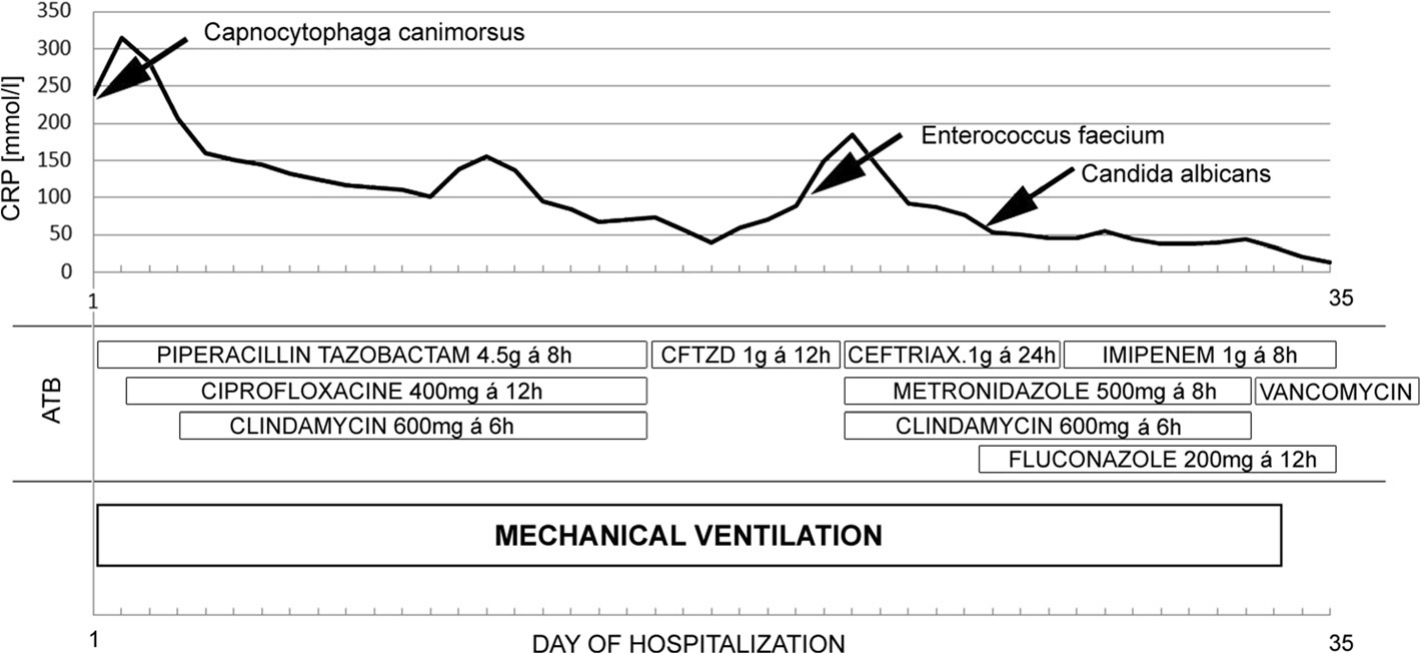
**Schema of antibiotics application during admission in ICU.** On the left side there is shown antibiotic treatment given for Capnocytophyga canimorsus infection (Piperacillin Tazobactam, Ciprofloxacine and Clindamycin), on the right side the change of antibiotics for Enterococcus faecium and Candida albicans superinfection (Ceftazidim, Ceftriax, Imipenem, Vancomycin, Fluconazole, Clindamycin). The arrows show time of positive microbiological examinations for mentioned microbiological species). Abbreviations in Figure 1 (ATB…Antibiotics, CRP…C reactive protein, CFTZD…Ceftazidim, mg …milligrams, g…grams).

However, before we could remove the tracheal tube the patient’s condition suddenly worsened, with a deterioration in respiratory function. She developed a fever, her CRP increased to 180 mg/L, procalcitonin to 2.54 μg/L and white blood cells to 22 × 10^9^/L. Clinical examination and a chest X-ray revealed a huge bilateral fluidothorax due to pleuropneumonia on both sides. Both pleural cavities were punctured and drained for a few following weeks. The daily output of pleural effusion reached 2–2.5 L. According to Light criteria, exudates on both sides had high protein and lactate dehydrogenase concentrations. Microscopic examination identified Gram-negative acid-resistant bacteria, but exudate cultivation was recurrently negative. She was empirically given another antibiotic (Figure 1).

Finally, bronchoscopy was performed and bronchoalveolar lavage microbiological examination revealed *Enterococcus faecium* at a concentration of 10^5^, which was sensitive to vancomycin, and *Candida albicans* at a concentration of 10^7^, which was sensitive to fluconazole. In the 2–3 weeks following vancomycin and fluconazole application, the patient gradually improved but pleural suction was necessary until the 60th day of admission. However, removal of the tracheal cannula was possible 10 days earlier.

The patient was successfully rehabilitated in the general ward and 103 days after admission, she was discharged home in good condition.

The described patient was one of the most susceptible individuals to *Capnocytophaga canimorsus* sepsis because she had a splenectomy. The spleen has a crucial role in prevention of bacterial infection because of production of IgM by B-lymphocytes and complement, and tuftsin activation participating in opsonization of Gram-negative bacilli [15]. The clinical picture of fully evolved sepsis by *Capnocytophaga canimorsus* appeared within 3–4 days as described in previously reported cases [4,5,7]. The course of disease was complicated by multiorgan failure – septic shock, ARDS, acute kidney failure, and disseminated coagulation. Fortunately, the patient improved after early empiric antibiotic treatment and vigorous support for vital functions, including artificial ventilation and CVVHD, and after 2 weeks we started weaning her from assisted ventilation.

However, 3 weeks after admission the patient again deteriorated and developed bilateral pleuropneumonia. A nosocomial infection was immediately suspected, and her antibiotic treatment was modified according to consultation with the hospital microbiology laboratory. This time *Capnocytophaga canimorsus* was no longer present but *Enterococcus faecium* and *Candida albicans* were revealed by bronchoalveolar lavage culture.

## Conclusion

The risk of severe sepsis due to *Capnocytophaga canimorsus* in patients after splenectomy is significantly higher than in the rest of the population [16]. Thus, some physicians recommend immediate prophylactic application of antibiotics after a dog bite [17,18]. The possibility of life-threatening nosocomial infection during a stay in the intensive care unit is described in our report and provides more evidence to support this approach.

We believe that people who care for dogs and cats should be informed about *Capnocytophaga canimorsus*. The immediate application of routine and relatively cheap antibiotic agents after suffering a dog bite may prevent sepsis development as well as subsequent complications. Such an approach should be considered particularly in high-risk persons [19]. The development of antibiotic resistance is negligible because of the low incidence of *Capnocytophaga* infection. We also believe the economic cost is acceptable as there is only a limited time to prevent full evolution of life-threatening disease in the prodromal phase.

### Consent

Written informed consent was obtained from the patient for publication of this report and any accompanying images. A copy of the written consent is available for review by the Editor-in-Chief of this journal.

## Abbreviations

APACHE: Acute physiology and chronic health evaluation; APTT: Activated partial tromboplastin time; ARDS: Acute respiratory distress syndrome; CRP: C-Reactive protein; CVVHD: Continuous venovenous hemodialysis; INR: International normalized ratio; PICC: Peripherally inserted central venous catheter; NT-proBNP: N-Terminal pro-brain natriuretic peptide; RIFLE: Risk, injury, failure, loss of kidney function; SOFA: Sequential organ failure assesment.

## Competing interest

On behalf of all authors, the corresponding author states there is no competing interest.

## Authors’ contributions

DM, JM and JH reviewed published data about *Capnocytophaga canimorsus*, took care of the patient during her stay in the intensive care unit and also participated in manuscript preparation and revision. OH and JCH wrote the manuscript. All authors read and approved the final manuscript.

## Supplementary Material

Additional file 1 Dog bite. Click here for file

Additional file 2 Chest x-ray in emergency room and after admission. Click here for file

Additional file 3 Petechiae due to thrombocytopenia. Click here for file
