# Supplementary figures and images for: Antibiotic treatment following a dog bite in an immunocompromized patient in order to prevent Capnocytophaga canimorsus infection: a case report
Source: BMC Res Notes. 2014 Jul 5;7:432. doi: 10.1186/1756-0500-7-432 (PMC4118785; doi:10.1186/1756-0500-7-432)

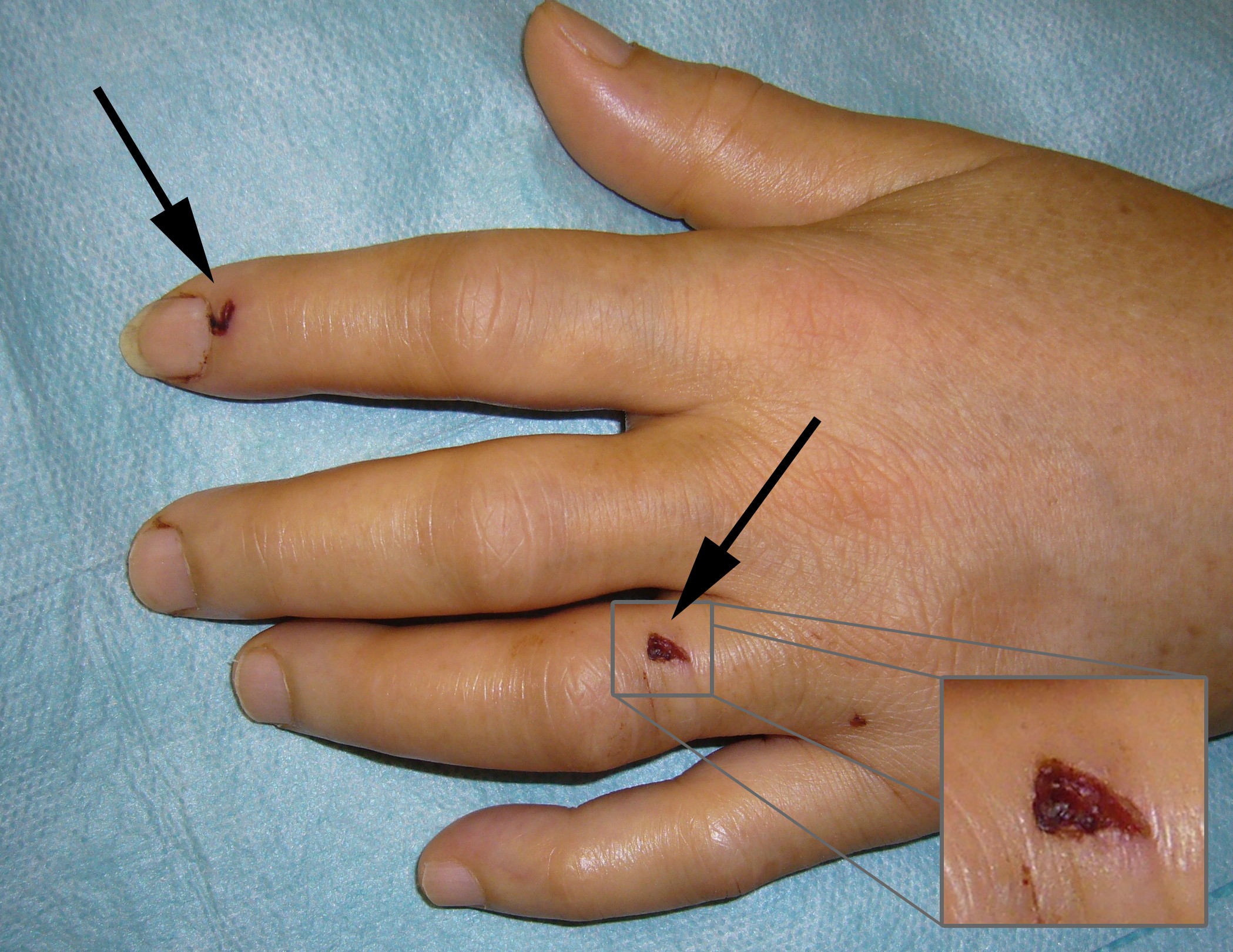

Supplement: Additional file 1 — Dog bite. [file 1756-0500-7-432-S1.jpeg]

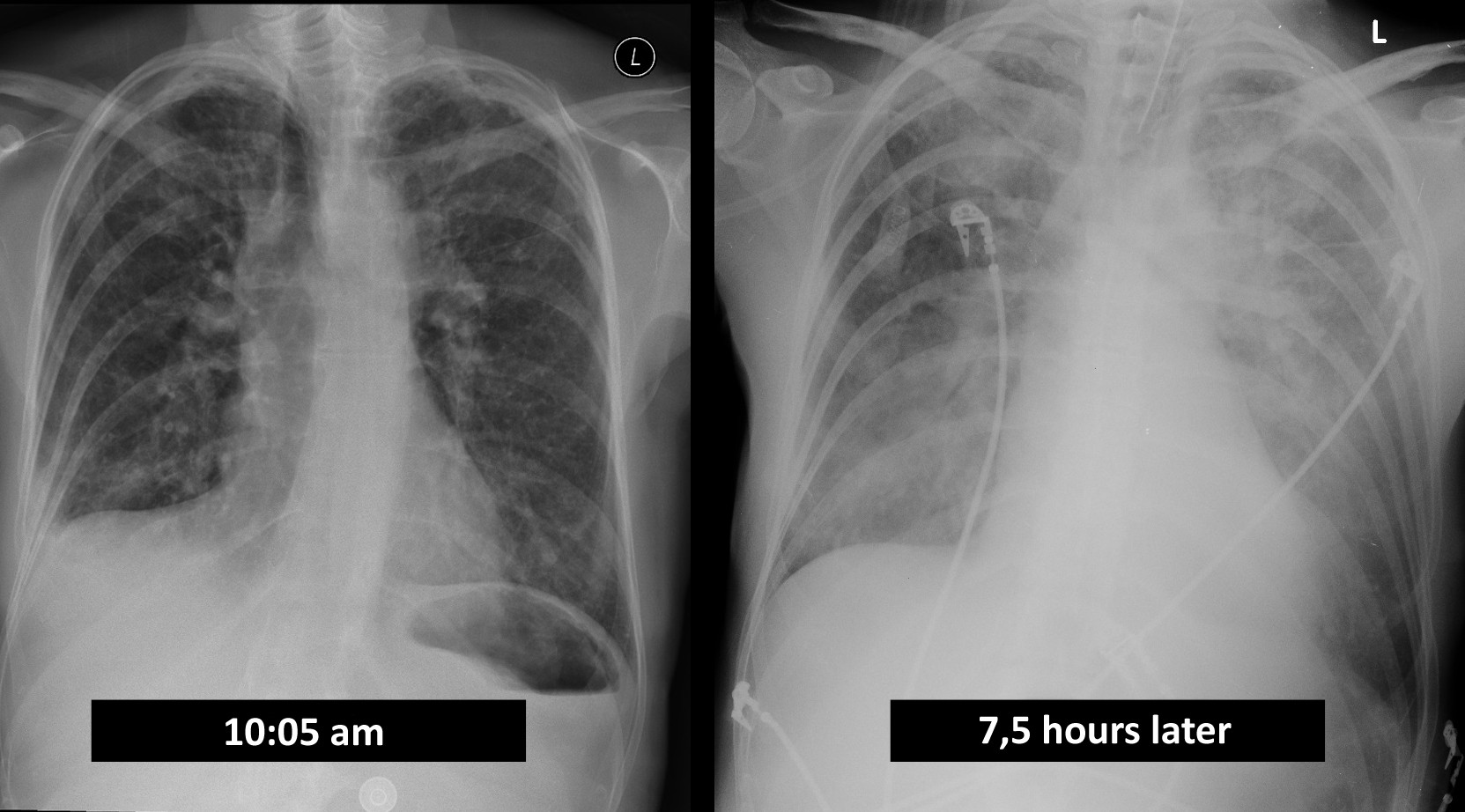

Supplement: Additional file 2 — Chest x-ray in emergency room and after admission. [file 1756-0500-7-432-S2.jpeg]

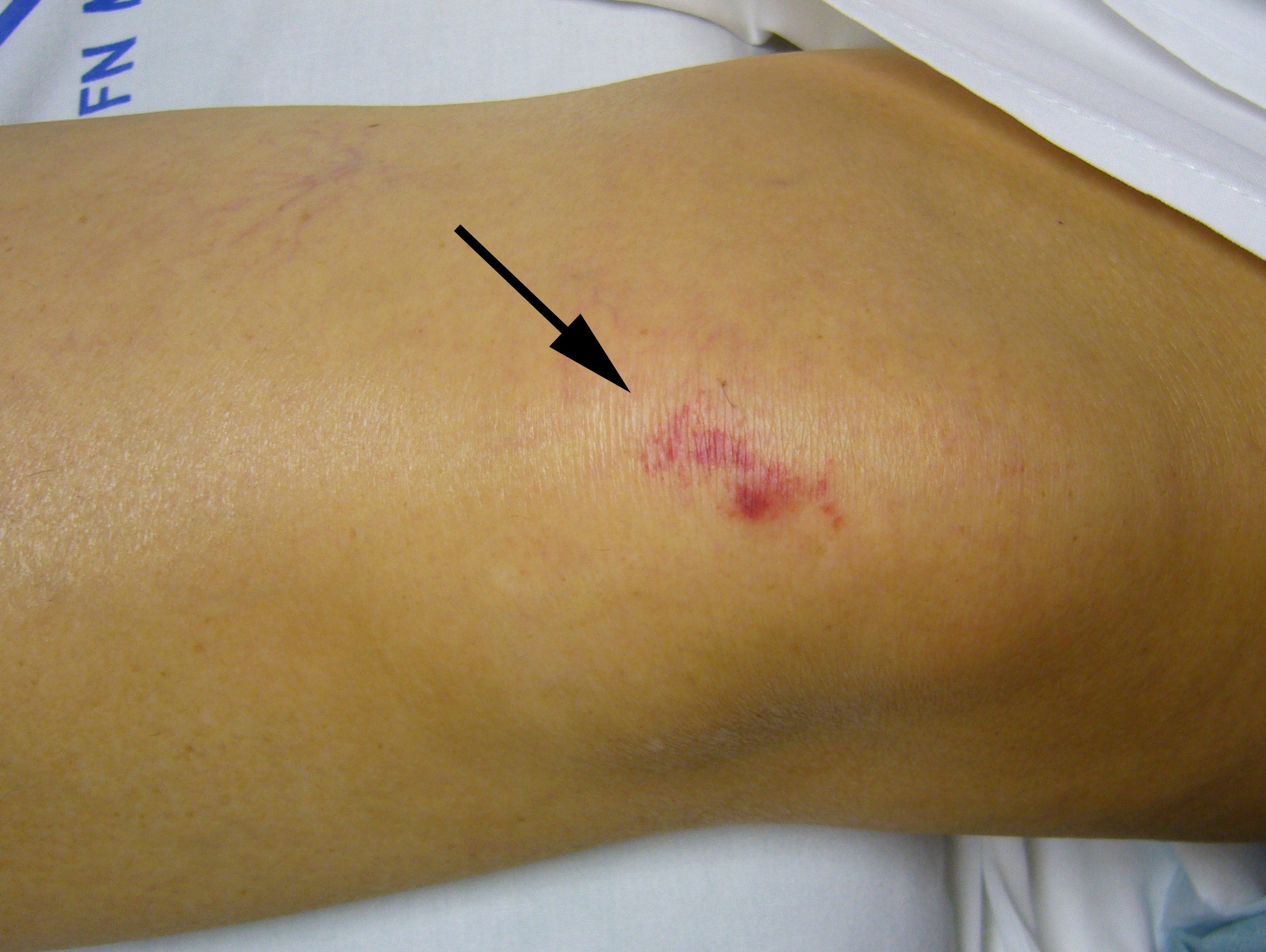

Supplement: Additional file 3 — Petechiae due to thrombocytopenia. [file 1756-0500-7-432-S3.jpeg]
